# Comparison of Polysaccharides from Two Species of *Ganoderma*

**DOI:** 10.3390/molecules17010740

**Published:** 2012-01-13

**Authors:** Jing Xie, Jing Zhao, De-Jun Hu, Jin-Ao Duan, Yu-Ping Tang, Shao-Ping Li

**Affiliations:** 1 State Key Laboratory of Quality Research in Chinese Medicine, University of Macau, Macao SAR 999078, China; 2 Institute of Chinese Medical Sciences, University of Macau, Macao SAR 999078, China; 3 Jiangsu Key Laboratory for TCM Formulae Research, Nanjing University of Chinese Medicine, Nanjing 210029, China

**Keywords:** *Lingzhi*, polysaccharides, HPSEC-ELSD, HPSEC-MALLS-RI, enzymatic digestion, HPTLC

## Abstract

*Ganoderma lucidum* and *Ganoderma sinense*, known as *Lingzhi* in Chinese, are commonly used Chinese medicines with excellent beneficial health effects. Triterpenes and polysaccharides are usually considered as their main active components. However, the content of triterpenes differs significantly between the two species of *Ganoderma*. To date, a careful comparison of polysaccharides from the two species of *Ganoderma* has not been performed. In this study, polysaccharides from fruiting bodies of two species of *Lingzhi* collected from different regions of China were analyzed and compared based on HPSEC-ELSD and HPSEC-MALLS-RI analyses, as well as enzymatic digestion and HPTLC of acid hydrolysates. The results indicated that both the HPSEC-ELSD profiles and the molecular weights of the polysaccharides were similar. Enzymatic digestion showed that polyshaccharides from all samples of *Lingzhi* could be hydrolyzed by pectinase and dextranase. HPTLC profiles of their TFA hydrolysates colored with different reagents and their monosaccharides composition were also similar.

## 1. Introduction

*Ganoderma*, known as “*Lingzhi*” in Chinese, is a genus of fungi belonging to the family Polyporaceae. Up to date, 98 species of *Ganoderma* have been found in China. However, only two species of *Ganoderma*, *G. lucidum* and *G. sinense*, are recorded in the Chinese Pharmacopoeia (2010). Triterpenes and polysaccharides are usually considered the main active components in *Lingzhi*, however, the content of triterpenes differs significantly between the two species of *Ganoderma* [1]. Modern pharmacologic studies have revealed that polysaccharides have multiple pharmacological activities [2,3,4]. Actually, the effects of polysaccharides are closely related to their chemical characteristics, such as monosacchride composition, molecular mass, configuration, and position of the glycoside linkages [5,6,7]. To date, no careful comparison of the polysaccharides from the two species of *Ganoderma* has been performed, though it was noticed that enzymatic hydrolysates of polysaccharides from two species of *Ganoderma* were different [8].

So far, high-performance liquid chromatography (HPLC), gas chromatography (GC), mass spectrometry (MS), nuclear magnetic resonance (NMR) [9,10] and high-performance thin-layer chromatography (HPTLC) [11,12] have been used for determination of chemical properties of polysaccharides from *Lingzhi*. However, almost all studies have focused on the polysaccharides of *G. lucidum* (GLP) rather than those of *G. sinense* (GSP). In this study, polysaccharides from the two species of *Lingzhi* collected from different regions of China were analyzed and compared. It is helpful to elucidate the characters of polysaccharides from *Lingzhi*, which is beneficial for quality control.

## 2. Results and Discussion

### 2.1. HPSEC-ELSD Profiles and Molecular Weights of Polysaccharides from Lingzhi

**Figure 1:**
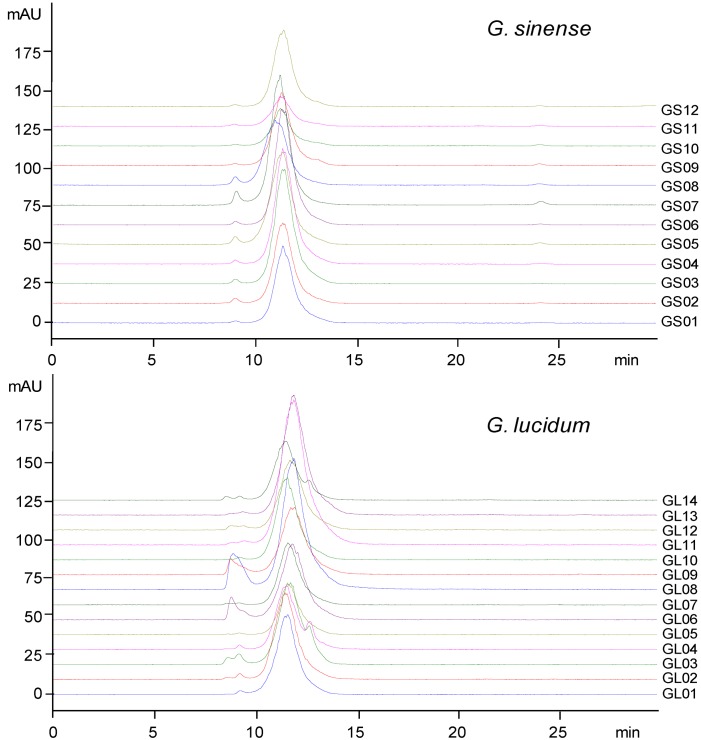
HPSEC-ELSD profiles of crude polysaccharides from *Lingzhi*.

**Figure 2:**
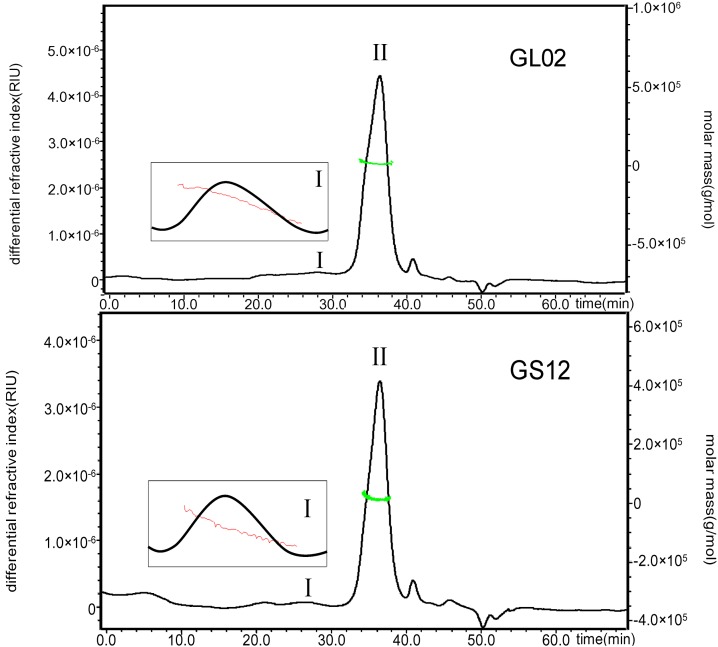
HPSEC-RI profiles with molecular weight distribution of polysaccharides from typical samples of *G. lucidum* (GL02) and *G. sinense* (GS12).

High performance size exclusion chromatography-evaporative light scattering detection (HPSEC-ELSD) profiles of crude polysaccharides from two species of *Ganoderma* used as *Lingzhi* are shown in Figure 1. The results indicated that polysaccharides from *G. lucidum* and *G. sinense* were similar, based on the retention time. Their molecular weights (M_w_) were estimated using HPSEC coupled with multi-angle laser light scattering (MALLS) and refractive index (RI) detection, which showed that the M_w_ values of peak I and peak II in GLP and GSP were 4.35 × 10^6^ (±0.7%) and 1.88 × 10^4^ (±11%), 7.08 × 10^6^ (±2%) and 1.53 × 10^4^ (±22%) g/mol, respectively. Peak I in both GLP and GSP had a wide polydispersity, but peak II showed a narrow molecular weight distribution (Figure 2). Actually, these polysaccharide fractions could also be different with varying monosaccharide compositions, ratios and glycosidic linkages.

### 2.2. Investigation on Enzymatic Digestion of Polysaccharides from Lingzhi

Enzymatic digestion, which has been used in the discrimination of polysaccharides from traditional Chinese medicines, is a specific and mild condition hydrolysis method with higher selectivity [8]. Previous studies showed that polysaccharides from *Lingzhi* usually consist of arabinose, galactose, glucose, xylose and mannose. Considering the linkages, (1→3)-β-D-glucosidic, (1→4)-β-D-glucosidic, (1→6)-β-D-glucosidic, and α-D-glucosidic ones exist in *Ganoderma* polysaccharides [13,14,15,16], so dextranase, pectinase, cellulase, β-mannanase, xylanase, lichenase and β-glucanase were selected for enzymatic digestion of polysaccharides from *Lingzhi*.

**Figure 3:**
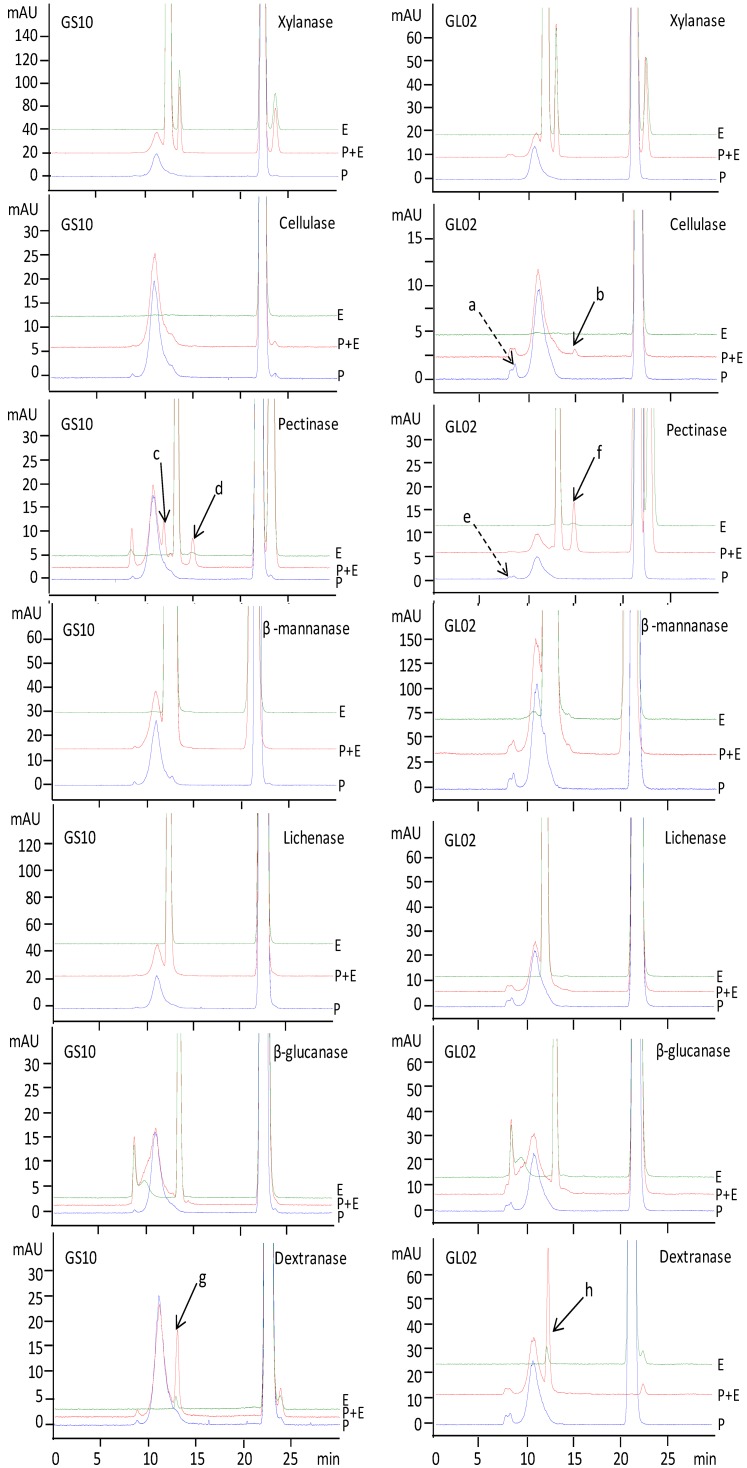
HPSEC-ELSD profiles of polysaccharides from *G. lucidum* (GL02) and *G. sinense* (GS10) treated with (**P+E**) or without(**P**) selected enzyme (**E**).

Representative ELSD profiles of polysaccharides from *G. lucidum* (GL02) and *G. sinense* (GS10) before and after digestion with selected glycoside hydrolases are shown in Figure 3. Enzyme solutions treated by the same procedure were used as controls.

The results showed that xylanase, β-mannanase, lichenase and β-glucanase usually had no significant effect on polysaccharides from *G. lucidum* (GL02) and *G. sinense* (GS10), but pectinase (peak c, peak d and peak f) and dextranase (peak g and peak h) can certainly hydrolyze polysaccharides from GS10 and GL02. Cellulase also had an effect on polysaccharides from GL02 (peak a decreased and peak b present). The detailed responses of polysaccharides from *G. sinense* and *G. lucidum* to enzymatic digestion are summarized in Table 1. Polysaccharides from all samples of *G. sinense* and *G. lucidum* showed positive responses to enzymatic digestion. In addition, some samples had specific responses to cellulase (GL02 and GL13) and β-mannanase (GL01, GL07, GL10, GL11 and GL14). 

**Table 1:** Responses of polysaccharides from *Lingzhi* to enzymatic digestion.

Polysaccharides	Enzymes						
	Xylanase	Cellulase	Pectinase	β-mannanase	Lichenase	β-glucanase	Dextranase
GS01	− ^a^	−	+	−	−	−	+
GS02	−	−	+	−	−	−	+
GS03	−	−	+	−	−	−	+
GS04	−	−	+	−	−	−	+
GS05	−	−	+	−	−	−	+
GS06	−	−	+	−	−	−	+
GS07	−	−	+	−	−	−	+
GS08	−	−	+	−	−	−	+
GS09	−	−	+	−	−	−	+
GS10	−	−	+	−	−	−	+
GS11	−	−	+	−	−	−	+
GS12	−	−	+	−	−	−	+
GL01	−	−	+	+	−	−	+
GL02	−	+	+	−	−	−	+
GL03	−	−	+	−	−	−	+
GL04	−	−	+	−	−	−	+
GL05	−	−	+	−	−	−	+
GL06	−	−	+	−	−	−	+
GL07	−	−	+	−	−	−	+
GL08	−	−	+	−	−	−	+
GL09	−	−	+	−	−	−	+
GL10	−	−	+	+	−	−	+
GL11	−	−	+	+	−	−	+
GL12	−	−	+	−	−	−	+
GL13	−	+	+	−	−	−	+
GL14	−	−	+	+	−	−	+

^a^ +, positive response; −, negative response.

### 2.3. Acid Hydrolysates of Polysaccharides from Lingzhi

#### 2.3.1. Optimization of Trifluoroacetic Acid (TFA) Hydrolysis

**Figure 4:**
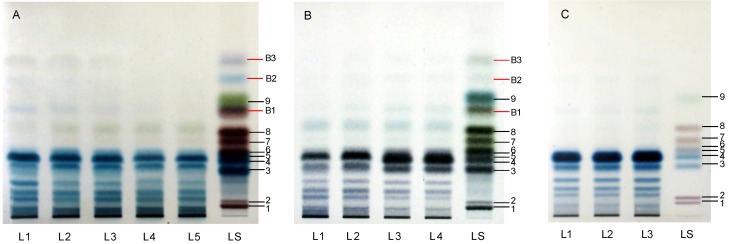
Effects of (**A**) concentration of TFA for 2 h, (**B**) hydrolysis time treated with 5 mol/L, and (**C**) optimum conditions (5 mol/L for 6 h) on acid hydrolysis of polysaccharides from *Ganoderma lucidum* (GL07). **A**: L1, 7 mol/L; L2, 6 mol/L; L3, 5 mol/L; L4, 4 mol/L; L5, 3 mol/L. **B**: L1, 2 h; L2, 4 h; L3, 6 h; L4, 8 h. **C**: L1, L2 and L3 were all at same optimum conditions. LS in **A** and **B**, mixed standards treated with 8 mol/L TFA for 2h; LS in **C**: Mixed standards of D-galacturonic acid (**1**), D-glucuronic acid (**2**), D-galactose (**3**), D-glucose (**4**), D-mannose (**5**), L-arabinose (**6**), D-xylose (**7**), D-ribose (**8**)and L-rhamnose (**9**).

HPTLC is a simple and effective tool for determination of mono-, di-, oligosaccharides [17,18], so it was employed to test the monosaccharide composition of polysaccharides from *G. lucidum* and *G. sinense*. Crude polysaccharides from *G. lucidum* (GL07) were used for optimization of TFA concentration and hydrolysis time. In brief, polysaccharides obtained from one gram of sample were hydrolyzed using different concentrations (3, 4, 5, 6, 7 and 8 mol/L) of TFA solution. A standard sugar mixture was also treated with high concentration TFA so as to know the effect of TFA on the stability of monosaccharides. Three additional bands (B1, B2 and B3) were found in standard sugar mixture treated with 8 mol/L TFA for two h (Figure 4A), but no additional bands appeared in standard sugar mixture without TFA treatment (Figure 4C). The results suggested that some monosaccharides could be degraded by high concentrations of TFA (L1 and L2 in Figure 4A). Therefore, the concentration of TFA for acid hydrolysis of polysaccharides should not be higher than 5 mol/L. In addition, complete acid hydrolysis is easily performed under higher concentration. Finally, 5 mol/L TFA was selected so as to ensure complete hydrolysis and avoid degradation of monosaccharides.

Acid hydrolysis time of polysaccharides was carried out in 5 mol/L TFA solution for 2, 4, 6 and 8 h. The blue bands with low Rf present in chromatograms of hydrolysis of the polysaccharides for 2 h and 4 h were very clear, which gradually disappeared as the hydrolysis time was extended. Similar chromatograms were obtained after hydrolysis for 6 h and 8 h (Figure 4B), which indicated 6 h was adequate for complete hydrolysis. Moreover, the sample of polysaccharides from GL07 was hydrolyzed in triplicates under optimized conditions to evaluate the repeatability of the acid hydrolysis. The result showed that TFA hydrolysis of polysaccharides had a good repeatability (Figure 4C).

#### 2.3.2. HPTLC Chromatograms of Monosaccharides and Protein Ingredients in Polysaccharides

**Figure 5:**
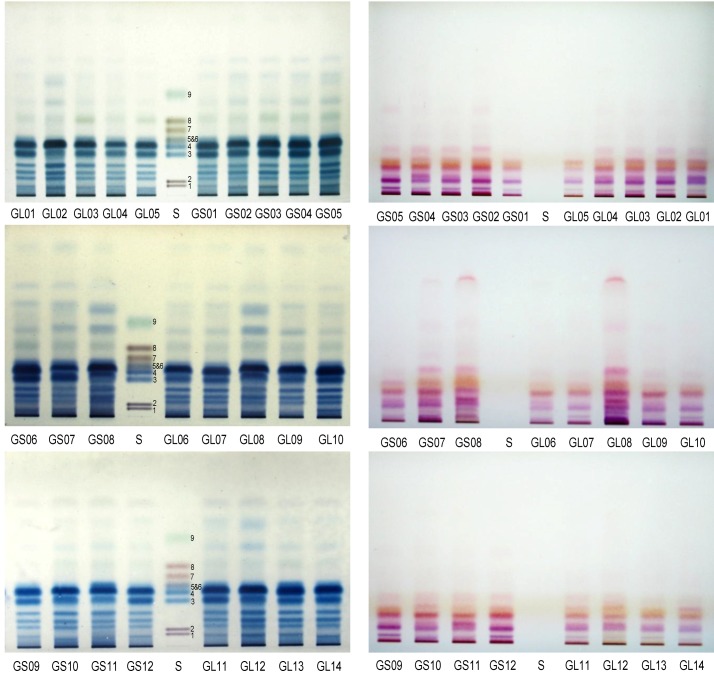
HPTLC profiles, colored with aniline-diphenylamine-phosphoric acid (Left) and ninhydrin (Right) solutions, of acid hydrolysates of polysaccharides from *Linzhi*. GL01-GL14 and GS01-GS12 were the same as in Section 3.1. **S**: Mixed standards of D-galacturonic acid (**1**), D-glucuronic acid (**2**), D-galactose (**3**), D-glucose (**4**), D-mannose (**5**), L-arabinose (**6**), D-xylose (**7**), D-ribose (**8**)and L-rhamnose (**9**).

HPTLC profiles of acid hydrolysates of crude polysaccharides from *G. lucidum* and *G. sinense* are shown in Figure 5, which was colored with aniline-diphenylamine-phosphoric acid solution and ninhydrin solution, respectively. The results suggested that both *G. lucidum* and *G. sinense* had similar saccharide profiles with obvious bands corresponding to galactose and glucose. It was reported that most polysaccharides from *Lingzhi* were glycoproteins or glycopeptides [19]. Therefore, ninhydrin solution was used for detection of amino acids. The chromatograms indicated that there was significant difference between the two species of *Ganoderma* used as *Lingzhi*.

## 3. Experimental

### 3.1. Herbal Materials and Chemicals

Different samples of *G. lucidum* (GL) and *G. sinense* (GS) were collected from nine Chinese provinces, *i.e.*, GS01-04 and GL01-05, GS05, GS06-07 and GL06-10, GS08, GS09-10, GS11 and GL13, GS12 and GL14, GL11, GL12 were from Anhui, Guangxi, Shandong, Guizhou, Macau, Hunan, Beijing, Zhejiang, Sichuan, respectively. The identities of the two species of *Ganoderma* were confirmed by Prof. Xiaolan Mao, Institute of Microbiology, Chinese Academy of Sciences. The voucher specimens of *Ganoderma* were deposited at the Institute of Chinese Medical Sciences, University of Macau, Macau, China.

Deionized water was prepared using a Millipore Milli Q-Plus system (Millipore, Billerica, MA, USA). HPLC grade methanol (Merck, Darmstadt, Germany) was used for sample preparation. D-Galacturonic acid (99%), D-glucuronic acid (99%), D-(−)-ribose (99%), D-(+)-xylose (99%), L-(+)-arabinose (99%), D-(+)-mannose(99%), L-rhamnose monohydrate (99%), D-(+)-galactose (99%), D-(+)-glucose (99%), phosphoric acid (85%), and ninhydrin (A.C.S. reagent) were purchased from Sigma–Aldrich (St. Louis, MO, USA). Cellulase (endo-1,4-β-D-glucanase, EC 3.2.1.4), pectinase (Polygalacturonanase, EC 3.2.1.15), dextranase (EC 3.2.1.11) and β-glucanase [endo-1,3(4)-β-glucanase, EC 3.2.1.6] were obtained from Sigma (St. Louis, MO, USA); xylanase (EC 3.2.1.8), β-mannanase (EC 3.2.1.78) and lichenase [endo-1,3(4)-β-D-glucanase, EC 3.2.1.73] were purchased from Megazyme (Wicklow, Ireland). All the other reagents were of analytical grade.

### 3.2. Preparation of Solutions

Standard sugar mixtures containing 0.5 mg/mL of glucose, rhamnose, mannose, 0.6 mg/mL of galactose, 1.0 mg/mL of arabinose, galacturonic acid, glucuronic acid, 1.5 mg/mL of ribose, xylose, were prepared in 95% ethanol. Aniline-diphenylamine-phosphoric acid solution was prepared by dissolving and mixing diphenylamine (4 g), aniline (4 mL), and 85% phosphoric acid (20 mL) in acetone (200 mL). Ninhydrin solution was prepared by dissolving ninhydrin (0.2 g) and mixing acetic acid (0.2 mL) in 100 mL absolute ethanol.

### 3.3. Preparation of Polysaccharides

The fruit bodies of *Lingzhi* were carefully cleaned and cut into slices, then dried at 40 °C for 12 h. Dried slices were pulverized and then passed through a 0.8 mm mesh. Sample materials (1.0 g) were immersed in water (30 mL) and refluxed in a Syncore parallel reactor (Büchi, Switzerland) for 1 h at 100 °C with stirring at 200 rpm, respectively. Then the extract solution was centrifuged at 4,500 × g for 10 min (Allegre X-15R centrifuge; Beckman Coulter, Fullerton, CA, USA). An aliquot of supernatant (20 mL) was evaporated to dryness under vacuum. The residue was dissolved in water (5 mL), then ethanol was added to a final concentration of 80% (v/v) for precipitation of crude polysaccharides. After standing for 6 h at 4 °C, centrifugation (4,000 × g for 10 min) was performed. The precipitate was dried on water bath (60 °C), and then redissolved in hot (60 °C) water (4 mL). After centrifugation, the supernatant was transferred to an ultracentrifugal filter (molecular weight cut-off: 3 kDa, Millipore, Billerica, MA, USA), and then the low molecular weight compounds were removed by centrifugation (3000 × g, 30 min, 25 °C) for three times. Finally, the crude polysaccharides, which were prepared in duplicates, were obtained for further analysis.

### 3.4. HPSEC-ELSD Analysis

Crude polysaccharides were dissolved in water (4 mL), and then centrifuged at 13,200 rpm for 5 min (5415D. Eppendorf, Hamburg, Germany). Each supernatant was analyzed on an Agilent 1100 series LC system (Agilent Technologies, Palo Alto, CA, USA) coupled with evaporative light scattering detector (ELSD-LTα. Shimadzu. Japan). A TSK G-3000PWXL column (300 mm × 7.8 mm, i.d., 10 μm, Tosoh Bioscience, Tokyo, Japan) was used at 30 °C with an injection volume of 10 μL for separation of polysaccharides. Isocratic elution was operated with 20 mmol/L ammonium acetate aqueous solution at a flow-rate of 0.6 mL/min. The parameters of ELSD were set as follows: The drift tube temperature was 50 °C and nebulizer nitrogen gas pressure was at 350 KPa.

### 3.5. HPSEC-MALLS-RI Analysis

Crude polysaccharides from *G. lucidum* (GL02) and *G. sinense* (GS12) were dried with a nitrogen evaporator and dissolved in initial mobile phase (4 mL). Sample solutions were filtered through 0.22 μm nylon syringe filter before test on an Agilent 1100 series LC system coupled with a DAWN EOS multi-angle laser light scattering photometer (Wyatt Technology Co., Santa Barbara, CA, USA) and RI detector (G1362A, Agilent Technologies Inc.). A sample of 50 μL was injected into the system, and separated at 40 °C on two TSK G-6000PWXL (300 mm × 7.8 mm, i.d., 10 μm, Tosoh Bioscience, Tokyo, Japan) and TSK G-3000PWXL columns connected in series columns. Isocratic elution was performed with 15 mmol/L sodium chloride aqueous solution at a flow-rate of 0.5 mL/min. The [dn/dc] value for the tested samples was given as 0.140 mL/g. The data and chromatograms were recorded and processed by using ASTRA software (Wyatt Technology Co). The DWAN EOS photometer was calibrated by using HPLC grade toluene (Merck) and normalized with a BSA standard (A9647, Sigma).

### 3.6. Enzymatic Digestion

The phenol-sulfuric acid assay was applied to quantify the concentration of polysaccharides to 1 mg/mL calculated as glucose [20]. For 200 µL enzymatic digestion system, it contains 100 µL polysaccharide solution, an enzyme (final concentration 5 U/mL), and optimum buffer (Table 2). The reaction was carried out for 12 h at 40 °C and stopped by heating for 1 h at 80 °C. After centrifugation (15700 × g) at 4 °C for 30 min (CT15RE, Hitachi Koki Co., Ltd.), the supernatant was used for HPSEC-ELSD analysis. Deionized water instead of polysaccharide solution, treated as mentioned above, was used as blank control. The enzymatic activities were detected before use.

**Table 2:** Digestion buffers for various enzyme digestion modified from the operation manual.

Enzyme	EC number	Buffer solution	PH
Xylanase	EC 3.2.1.8	25 mM sodium acetate	4.7
Cellulase	EC 3.2.1.4	25 mM sodium acetate	4.5
Pectinase	EC 3.2.1.15	50 mM sodium acetate	5.5
β-mannanase	EC 3.2.1.78	50 mM sodium acetate	4.5
Lichenase	EC 3.2.1.73	25 mM sodium phosphate	6.5
β-glucanase	EC 3.2.1.6	50 mM sodium acetate	6.0
Dextranase	EC 2.1.1.11	50 mM sodium acetate	5.0

### 3.7. Acid Hydrolysis for Crude Polysaccharides

The crude polysaccharides were mixed with 5 mol/L TFA solution (3 mL) in a reaction tube and refluxed in a Syncore parallel reactor (Büchi, Switzerland) for 6 h at the temperature of 100 °C. After cooling, the hydrolysate was evaporated to dryness with a nitrogen evaporator at 55 °C. Ethanol (50%, 1 mL) was then added to dissolve the hydrolysate, and insoluble residue was removed by centrifugation (13,200 rpm, 5 min), and the supernatant was finally analyzed by HPTLC.

### 3.8. HPTLC Procedures

All the samples were applied on 0.2 mm nano-silica gel 60 HPTLC plates (Macherey–Nagel, Düren, Germany) with an AS30 HPTLC applicator (Dessaga, Germany). The bands were 10 mm wide, 16 mm distance, and 10 mm from the bottom edge. In order to optimize the acid condition, acid hydrolyzates of standard sugar mixture and polysaccharides from *G. lucidum* (GL07) (10 μL) were applied to the plates. For the repeatability evaluation of acid hydrolysis, the acid hydrolysates of polysaccharides from *Lingzhi* (10 μL) and mixed standards (1 μL) were applied to the plates. Then all the plates were developed to a distance of 90 mm with chloroform–*n*-butanol–methanol–acetic acid–water 5.5: 11.0: 5.0: 1.5: 2.0 (v/v) as mobile phase at room temperature (around 25 °C). The developed plates were colorized with aniline–diphenylamine–phosphoric acid solution and heated at 130 °C for 10 min or sprayed with ninhydrin solution and heated at 105 °C for 10 min, to make bands colored clearly. Then the plates were covered with transparent glasses and photographed.

## 4. Conclusions

In this study, crude polysaccharides from *G. lucidum* and *G. sinense*, were analyzed and compared. The results indicated that both the HPSEC-ELSD profiles and the molecular weights of the polysaccharides were similar. Enzymatic digestion showed that polyshaccharides from all samples of *Lingzhi* could be hydrolyzed by pectinase and dextranase. HPTLC profiles of their TFA hydrolysates colored with different reagents and their monosaccharide composition were also similar. Considering the resolution of HPTLC, further investigation is need. 

## References

[1] Zhao J., Zhang X.Q., Li S.P., Yang F.Q., Wang Y.T., Ye W.C. (2006). Quality evaluation of *Ganoderma through* simultaneous determination of nine triterpenes and sterols using pressurized liquid extraction and high performance liquid chromatography. J. Sep. Sci..

[2] Chen H.S., Tsai Y.F., Lin S., Lin C.C., Khoo K.H., Lin C.H., Wong C.H. (2004). Studies on the immuno-modulating and anti-tumor activities of *Ganoderma lucidum* (Reishi) polysaccharides. Bioorg. Med. Chem..

[3] Wang Y.Y., Khoo K.H., Chen S.T., Lin C.C., Wong C.H., Lin C.H. (2002). Studies on the immuno-modulating and antitumor activities of *Ganoderma lucidum* (Reishi) polysaccharides: functional and proteomic analyses of a fucose-containing glycoprotein fraction responsible for the activities. Bioorg. Med. Chem..

[4] Paterson R.R. (2006). *Ganoderma*—A therapeutic fungal biofactory. Phytochemistry.

[5] Tzianabos O. (2000). Polysaccharide immunomodulators as therapeutic agents: Structural aspects and biologic function. Clin. Microbiol. Rev..

[6] Pérez Q., Rodriguez-Carvajal M.A., Doco T. (2003). A complex plant cell wall polysaccharide: rhamnogalacturonan II. A structure in quest of a function. Biochimie.

[7] Leung M.Y.K., Liu C., Koon J.C.M., Fung K.P. (2006). Polysaccharide biological response modifiers. Immunol. Lett..

[8] Guan J., Li S.P. (2010). Discrimination of polysaccharides from traditional Chinese medicines using saccharide mapping—Enzymatic digestion followed by chromatographic analysis. J. Pharm. Biomed. Anal..

[9] Chang Y.W., Lu T.J. (2004). Molecular characterization of polysaccharides in hot-water extracts of *Ganoderma lucidum* fruitining bodies. J. Food Drug Anal..

[10] Huang S.Q., Li J.W., Li Y.Q., Wang Z. (2011). Purification and structural characterization of a new water-soluble neutral polysaccharide GLP-F1-1 from *Ganoderma lucidum*. Int. J. Biol. Macromol..

[11] Xin D., Kelvin K.C.C., Hei W.L., Carmen W.H. (2003). Fingerprint profiling of acid hydrolyzates of polysaccharides extracted from fruiting bodies and spores of *Lingzhi* by high-performance thin-layer chromatography. J. Chromatogr. A.

[12] Yang C., Guan J., Zhang J.S., Li S.P. (2010). Use of HPTLC to differentiate among the crude polysaccharides in six traditional Chinese medicine. JPC-J. Planar. Chromat..

[13] Lin Z.B. (2007). Modern Study of Lingzhi.

[14] Evsenko M.S., Shashkov A.S., Avtonomova A.V., Krasnopolskaya L.M., Usov A.I. (2009). Polysaccharides of basidiomycetes. alkali-soluble polysaccharides from the mycelium of white rot fungus *Ganoderma lucidum* (Curt.: Fr.) P. Karst. Biochemistry.

[15] Xu J., Liu W., Yao W.B., Pang X.B., Yin D.K., Gao X.D. (2009). Carboxymethylation of a polysaccharide extracted from *Ganoderma lucidum* enhances its antioxidant activities *in vitro*. Carbohyd. Polym..

[16] Ye L.B., Zhang J.S., Zhou K., Yang Y., Zhou S., Jia W., Hao R.X., Pan Y.J. (2008). Purification, NMR Study and Immunostimulating Property of a Fucogalactan from the Fruiting Bodies of *Ganoderma lucidum*. Planta Med..

[17] Doner L.W. (2011). Dertermining sugar composition of food gum polysaccharides by HPTLC. Chromatographia.

[18] Kyoko K., Toshiko U., Yasuyo O. (1985). Analyses of homogeneous D-gluco-oligosaccharides and -polysaccharides (degree of polymerization up to about 35) by high-performance liquid chromatography and thin-layer chromatography. J. Chromatogr. A.

[19] Ye L.B., Zhang J.S., Ye X.J., Tang Q.J., Liu Y.F., Gong C.Y., Dua X.J., Pand Y.J. (2008). Structural elucidation of the polysaccharide moiety of a glycopeptide (GLPCW-II) from *Ganoderma lucidum* fruiting bodies. Carbohyd. Res..

[20] Dubois M., Gilles K.A., Hamilton J.K., Rebers P.A., Smith F. (1956). Colorimetric method for determination of sugars and related substances. Anal. Chem..

